# Classification of Alzheimer's Disease, Mild Cognitive Impairment, and Cognitively Unimpaired Individuals Using Multi-feature Kernel Discriminant Dictionary Learning

**DOI:** 10.3389/fncom.2017.00117

**Published:** 2018-01-09

**Authors:** Qing Li, Xia Wu, Lele Xu, Kewei Chen, Li Yao, Alzheimer's Disease Neuroimaging Initiative

**Affiliations:** ^1^Department of Electronics, College of Information Science and Technology, Beijing Normal University, Beijing, China; ^2^State Key Laboratory of Cognitive Neuroscience and Learning, Beijing Normal University, Beijing, China; ^3^Banner Alzheimer's Institute and Banner Good Samaritan PET Center, Phoenix, AZ, United States

**Keywords:** Alzheimer's disease (AD), mild cognitive impairment (MCI), multimodal imaging, multiple kernel dictionary learning

## Abstract

Accurate classification of either patients with Alzheimer's disease (AD) or patients with mild cognitive impairment (MCI), the prodromal stage of AD, from cognitively unimpaired (CU) individuals is important for clinical diagnosis and adequate intervention. The current study focused on distinguishing AD or MCI from CU based on the multi-feature kernel supervised within-Class-similar discriminative dictionary learning algorithm (MKSCDDL), which we introduced in a previous study, demonstrating that MKSCDDL had superior performance in face recognition. Structural magnetic resonance imaging (sMRI), fluorodeoxyglucose (FDG) positron emission tomography (PET), and florbetapir-PET data from the Alzheimer's Disease Neuroimaging Initiative (ADNI) database were all included for classification of AD vs. CU, MCI vs. CU, as well as AD vs. MCI (113 AD patients, 110 MCI patients, and 117 CU subjects). By adopting MKSCDDL, we achieved a classification accuracy of 98.18% for AD vs. CU, 78.50% for MCI vs. CU, and 74.47% for AD vs. MCI, which in each instance was superior to results obtained using several other state-of-the-art approaches (MKL, JRC, mSRC, and mSCDDL). In addition, testing time results outperformed other high quality methods. Therefore, the results suggested that the MKSCDDL procedure is a promising tool for assisting early diagnosis of diseases using neuroimaging data.

## Introduction

Alzheimer's disease (AD) is a complex multifactorial neurodegenerative disorder and is the most common type of dementia, defined by extensive neuronal and synapses loss (Tan et al., 2013; Gao et al., 2016). Recent study has shown that AD has high prevalence of an estimated 40 million patients worldwide (Selkoe and Hardy, 2016). Mild cognitive impairment (MCI) has been generally viewed as an intermediate state between normal aging and the onset of AD (Petersen et al., 2001; Garcés et al., 2014). Thus, AD and MCI, the transitional stage between the healthy aging and dementia, which commonly characterized by slight cognitive deficits but largely intact activities of daily living (Petersen, 2004; Wei et al., 2016), have been greatly interested.

**Graphical Abstract:**
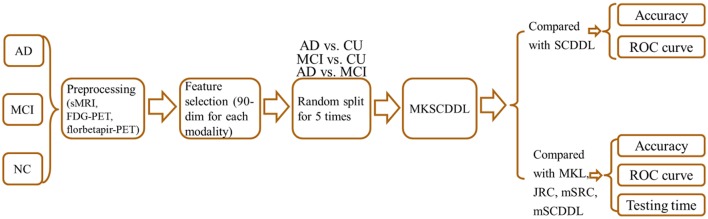


It has been shown that the neuroimaging data, including structural magnetic resonance imaging (sMRI) (Wee et al., 2011; Zhou et al., 2011), functional MRI (fMRI) (Suk et al., 2013), fluorodeoxyglucose positron emission tomography (FDG-PET) (Sanabria-Diaz et al., 2013), and amyloid PETs, such as Pittsburgh compound B (PiB-PET) (Zhang et al., 2014), florbetapir-PET (Saint-Aubert et al., 2013), can be used to discriminate AD or MCI with promising results when each modality is used individually and separately. It has been speculated that different neuroimaging tool provides complementary information, which, when combined, can be more powerful for diagnosis of AD or MCI (Liu et al., 2014b; Suk et al., 2015; Wang et al., 2016) and combining these potentially complementary information from various modalities would produce more powerful classifiers (Zhang et al., 2012a; Xu et al., 2015).

Several classification methods of combining multi-modality data have been used to classify AD or MCI from CU. For example, a weighted multiple kernel learning (MKL) model has been proposed to classify AD or MCI based on combining different modalities (Wee et al., 2012; Zhang et al., 2012b; Liu et al., 2014b). A joint regression and classification (JRC) algorithm was also introduced and has been indicated to diagnosis AD or MCI effectively based on multi-modalities data (Zhu et al., 2014a,b). A weighted multi-modality sparse representation-based classification (mSRC) was developed and applied for discriminating AD or MCI based on multi-modalities (Xu et al., 2015). Recently, a multi-modal discriminative dictionary learning (mSCDDL) (Li et al., 2017) algorithm has been proposed for classifying AD or MCI efficiently, which was a weighted multi-modality way extended from supervised within-Class-similarity discriminative dictionary learning (SCDDL), a robust and efficient machine learning method for facial recognition by Xu et al (Xu et al., 2016).

SCDDL was a discriminant dictionary learning (DL), which combined the classification error term and the within-Class-similarity in the objection function of DL scheme (Xu et al., 2016). Recently, SCDDL was extended to a kernel framework, due to MKL algorithm has been suggested to be effective for feature fusion (Gönen and Alpaydin, 2011), named as multi-feature kernel SCDDL (MKSCDDL) and has been indicated to be an efficient tool in face recognition (Wu et al., 2017).

In this study, MKSCDDL was examined for its robustness and efficiency of classification accuracy for AD or MCI with CU, based on three modalities data i.e., sMRI, FDG-PET and florbetapir-PET. Our experimental results indicated that the MKSCDDL method combined multi-modalities could outperform SCDDL with each modality data alone, and achieve better or comparable classification performance, compared with some other state-of-the-art multi-modality classification algorithms, including MKL (Zhang et al., 2011), JRC (Zhu et al., 2014a), mSRC (Xu et al., 2015), and mSCDDL (Li et al., 2017).

## Image preprocessing

In this work, we used data from the Alzheimer's Disease Neuroimaging Initiative (ADNI) for performance evaluation. The ADNI was launched in 2003 by the National Institute on Aging (NIA), the National Institute of Biomedical Imaging and Bioengineering (NIBIB), the Food and Drug Administration (FDA), private pharmaceutical companies, and non-profit organizations, as a 5-year public-private partnership. For up-to-date information, see http://www.adni-info.org.

### Subjects

In this paper, 113 patients with AD, 110 patients with MCI and 117 CU with the age ranged from 55 to 99 years were included. All the data, including the sMRI, FDG-PET, and florbetapir-PET, were downloaded from ADNI 1, ADNI GO, or ADNI 2. For each subject, the data-acquisition interval of the three modalities was within four months. Moreover, the subjects were matched in terms of age, the years of education and gender. The subjects we selected satisfied the following criteria: (1) The MMSE score of each AD subject was between 20 and 26, with a CDR of 0.5 or 1.0. The AD group did not significantly differ with respect to the presence of APOE4 alleles from the MCI group (*p* = 0.765), but had significantly lower MMSE scores (compared with CU group, *p* = 1.24 × 10^−90^; MCI group, *p* = 1.61 × 10^−40^) and a different presence of APOE4 alleles compared with the CU group (*p* = 0.014). (2) The MMSE score of each MCI subject was between 24 and 30, and the CDR was 0.5. The MCI group had significantly lower MMSE scores (*p* = 4.69 × 10^−31^) and a different presence of APOE4 alleles (*p* = 7.34 × 10^−04^) compared with CU group. (3) The MMSE score of each CU was between 26 and 30 and their CDR was 0.0. Table 1 shows the demographic information of the subjects.

**Table 1:** Demographic information of the subjects, *p*-value was obtained using one-way ANOVA to the AD, MCI, and CU groups.

	**AD (*n* = 113)**	**MCI (*n* = 110)**	**CU (*n* = 117)**	***p*-value**
Gender	62M/51F	59M/51F	62M/55F	0.96
Age	75.6 ± 7.6	75.2 ± 7.8	75.4 ± 7.0	0.94
EDU	16.10 ± 3.00	16.57 ± 2.76	16.44 ± 2.41	0.65
MMSE	22.4 ± 2.2	27.4 ± 1.9	28.9 ± 1.3	7.75 × 10^−75^
CDR	0.8 ± 0.2	0.5 ± 0.0	0.0 ± 0.0	7.00 × 10^−151^
APOE4 (%)	50.00	52.73	24.49	

*AD, Alzheimer's disease; MCI, mild cognitive impairment; CU, cognitively unimpaired; M, male; F, female; MMSE, Mini-Mental State Examination; CDR, Clinical Dementia Rating; EDU, years of education; APOE4, percentage of APOE4 alleles*.

### Image processing

Images were preprocessed using the VBM8 (Voxel-Based Morphometry 8) Toolbox (http://dbm.neuro.uni-jena.de/vbm8/) in SPM8 (Statistical Parametric Mapping 8) (http://www.fil.ion.ucl.ac.uk/spm/) that running on MATLAB 2010b (The MathWorks, Inc., Sherborn, MA, USA). Based on adaptive maximum posterior and partial volume estimation, every structural image was segmented into rigid-body-aligned gray matter (GM), white matter (WM) and cerebrospinal fluid (CSF) for each subject (Rajapakse et al., 1997; Tohka et al., 2004). Spatially adaptive non-local approach was applied to improve the segmentation. The diffeomorphic anatomical registration through exponential lie algebra (DARTEL) protocol (Ashburner, 2007) in which template creation and image registration were performed to normalize the gray-matter images iteratively by using a diffeomorphic anatomical registration.

All FDG-PET and florbetapir-PET images were co-registered with each individual's sMRI using a rigid body transformation, and subsequently warped to the cohort-specific DARTEL template. Then, the standard uptake value ratio (SUVr) image was calculated for each FDG-PET image and florbetapir-PET image; reference masks for quantification were defined relative to the whole brain (Langbaum et al., 2009; Sabbagh et al., 2015) or cerebellum (Reitan, 1958; Camus et al., 2012), respectively.

Then, based on the Automated Anatomical Labeling (AAL) (Tzourio-Mazoyer et al., 2002), 90 regions of interest (ROIs) (45 for each hemisphere; Table S1) were obtained. The feature of sMRI, FDG-PET, and florbetapir-PET were got by averaging the corresponding value of mean volume of GM, SUVr values of FDG-PET and florbetapir-PET from each ROI that all the voxels within the ROI of each subject.

## Method

### Discriminant dictionary learning

Suppose *n* training samples with *d*-dimension from *k* classes are represented by $A=\left[a_{1},a_{2},\ldots ,a_{n}\right]=\left[A_{1},\ldots ,A_{l},\ldots ,A_{k}\right]\in {ℜ}^{d\times n}$, in which, column vector *a*_*i*_ is the sample *i* (*i* = 1, …, *n*), and submatrix *A*_*j*_ consists of column vectors (samples) from class *j* (*j* = 1, …, *k*), and there are *m* atoms (each column of the dictionary can be viewed as an atom) in the corresponding dictionary $D=\left[d_{1},d_{2},\ldots ,d_{m}\right]\;\in {ℜ}^{d\times m}\left(m\leq n\right)$. The general supervised DL model can be denoted as follows:

(1) $$\begin{array}{lll} \langle D,\theta ,X\rangle & = & \text{arg}\underset{D,\theta ,X}{\text{min}}||A-DX|{|}_{F}^{2}+{\lambda_{1}||X||}_{1}+\lambda_{\theta }g\left(\theta \right)\\ s.t.||d_{j}|{|}_{2}^{2} & = & 1,\;for\;all\;j=1,\ldots ,m \end{array}$$

where θ is the discriminative parameter and *g*(θ) represents the discriminative term, *X* denotes the coding coefficients of training samples *A* on the dictionary *D*. *g*(θ) here indicates the linear classification error function (like $||H-WX|{|}_{F}^{2}$ in the DL methods of D-KSVD (Zhang and Li, 2010) and LC-KSVD (Jiang et al., 2013), where *H* is the class label matrix and *W* is a classifier).

For classification, the classifier learned with the dictionary may be optimal simultaneously, as in the DL algorithms that incorporate a linear classification error term (Zhang and Li, 2010). However, the inner-structure of representation coefficients between classes has not been considered in such approach. To further enhance the discriminant power of the dictionary, both the linear classifier and the direct restriction of within-Class scatter on coding coefficients in the above discriminant DL scheme in our previous study are indicated (Xu et al., 2016), which is referred to as the SCDDL algorithm.

### Supervised within-class-similar discriminative dictionary learning

Suppose $A=\left[A_{1},\ldots ,A_{l},\ldots ,A_{k}\right]\in {ℜ}^{d\times n}\;$denotes the *n d*-dimensional training samples from *k* classes, *D* ∈ ℜ^*d*×*m*^(*m* ≤ *n*) is the discriminative dictionary with *m* atoms that needs to be derived, and *X* represents the coding coefficients of training samples *A* on the dictionary *D*, denoted as $X=\left[X_{1},\ldots ,X_{l},\ldots ,X_{k}\right]\in {ℜ}^{m\times n}$, same as above. The SCDDL model can be written as follows:

(2) $$\begin{array}{lll} \langle D,W,X\rangle & = & \arg\underset{D,W,X}{\text{min}}||A-DX|{|}_{F}^{2}+{\alpha ||H-WX||}_{F}^{2}+\beta ||W|{|}_{F}^{2}\\ +\;{\lambda_{1}||X||}_{1}+\lambda_{2}\overset{k}{\underset{i=1}{\sum }}\left(||X_{i}-M_{i}|{|}_{F}^{2}+\eta ||X_{i}|{|}_{F}^{2}\right)\\ s.t.||d_{j}|{|}_{2}^{2} & = & 1,\;for\;all\;j=1,\ldots ,m \end{array}$$

where $||\cdot |{|}_{F}^{2}$ represents the Frobenius norm. ${∥A-DX∥}_{F}^{2}$ is the reconstructed error term of the training samples *A* on the newly constructed dictionary *D*, $\alpha {∥H-WX∥}_{F}^{2}+\beta {∥W∥}_{F}^{2}$ is the linear classification error term, and $\overset{k}{\underset{i=1}{\sum }}\left({∥X_{i}-M_{i}∥}_{F}^{2}+\eta {∥X_{i}∥}_{F}^{2}\right)$ is the within-Class-similar term. *W* ∈ ℜ^*k*×*m*^ is the parameter of the classifier; each column of *H* ∈ ℜ^*k*×*m*^ is a vector, corresponds to one training sample with the form as [0, 0, …, 1, …, 0, 0] ∈ ℜ^*k*^, where 1 locates the corresponding class of the training sample; and each column of *M*_*i*_ is the mean vector of the coefficients *X*_*i*_ corresponding to class *i*. According to the elastic-net theory, the term ${∥X_{i}∥}_{F}^{2}$ combined with the term ∥*X*∥_1_ might make the solution of Equation (2) more stable (Zou and Hastie, 2005); and η is set as η = 1 for simplicity (Yang et al., 2014). Then Equation (2) can be written as:

(3) $$\begin{array}{lll} \langle D,W,X\rangle & = & \arg\underset{D,W,X}{\text{min}}||A-DX|{|}_{F}^{2}+{\alpha ||H-WX||}_{F}^{2}+\beta ||W|{|}_{F}^{2}\\ +\;{\lambda_{1}||X||}_{1}+\lambda_{2}\overset{k}{\underset{i=1}{\sum }}\left(||X_{i}-M_{i}|{|}_{F}^{2}+||X_{i}|{|}_{F}^{2}\right)\\ s.t.||d_{j}|{|}_{2}^{2} & = & 1,\;for\;all\;j=1,\ldots ,m \end{array}$$

The optimization process of Equation (3) has been discussed in our previous study (Xu et al., 2016). In SCDDL, the directly restricted within-Class-similar term makes the coding coefficients similar within one class and the linear classification error term selects the optimal classifier. This combination has been shown to improve the discriminative classification of the dictionary (Xu et al., 2016).

After obtaining the dictionary *D* and classifier *W* in the SCDDL model, the test samples can be finally classified.

For a given test sample *y*, the representation coefficient on *D* is:

(4) $$\begin{array}{l} x=\arg\underset{x}{min\;}||y-D_{x}|{|}_{2}^{2}+\lambda ||x|{|}_{1} \end{array}$$

where λ is a scalar constant. The representation coefficient *x* can be simply combined with the linear classifier *W*. Then the final identification of the test sample *y* is obtained in the DL procedure with:

(5) $$\begin{array}{l} label\left(y\right)=\arg\underset{l}{\max}{\left\{W_{x}\right\}}_{l},l=1,2,\ldots ,k \end{array}$$

where {·}_*l*_ represents the *l*-th element in the brace, *x* contains discriminant information for classification.

### Multi-feature kernel SCDDL (MKSCDDL)

The SCDDL model is extended to a kernel framework for the further multi-feature fusion in our previous study (Wu et al., 2017). Suppose ϕ(·) is a mapping function from *R*^*N*^ to a higher dimensional feature space. To avoid the explicit high-dimensional mapping procedure, mercer kernels could be helpful. The common mercer kernels include the linear kernel *k*(*x, y*) = 〈*x, y*〉, which equals to non-mapping; the Gaussian kernels $k\left(x,y\right)=exp\left(-\frac{||x-y|{|}^{2}}{c}\right)$; the polynomial kernels *k*(*x, y*) = (〈*x, y*〉 + *c*)^*d*^ (*c* and *d* are parameters) and the sigmoid kernels *k*(*x, y*) = *tanh*(*a*(*x*^*T*^*y*) + *r*) (*a* and *r* are parameters) (Manevitz and Yousef, 2001; Hussain et al., 2011; Liu et al., 2013; Pham and Pagh, 2013; Dyrba et al., 2015).

The training samples *A* and dictionary *D* can be mapped to a higher dimensional space by a function of ϕ(·), then *A* and *D* in the SCDDL model can be replaced by $\phi \left(A\right)\in R^{d_{map}\times n}$ and $\phi \left(D\right)\in R^{d_{map}\times m}$ (*d*_*map*_ is the dimensional number in the mapping space) respectively for the kernel SCDDL framework as follows:

(6) $$\begin{array}{lll} \langle D,W,X\rangle & = & \arg\underset{D,W,X}{\text{min}}||\phi \left(A\right)-\phi \left(D\right)X|{|}_{F}^{2}+{\alpha ||H-WX||}_{F}^{2}\\ +\;\beta ||W|{|}_{F}^{2}+\;{\lambda_{1}||X||}_{1}\\ +\;\lambda_{2}\overset{k}{\underset{i=1}{\sum }}\left(||X_{i}-M_{i}|{|}_{F}^{2}+||X_{i}|{|}_{F}^{2}\right)\\ s.t.||d_{j}|{|}_{2}^{2} & = & 1,\;for\;all\;j=1,\ldots ,m \end{array}$$

The dictionary can be represented by the training samples as Equation (7), according to the represented theorem (Schölkopf et al., 2001):

(7) $$\begin{array}{l} \phi \left(D\right)=\phi \left(A\right)V \end{array}$$

where *V* ∈ *R*^*n*×*m*^ is the representation matrix. Equation (6) can be transformed to Equation (8) with Equation (7):

(8) $$\begin{array}{l} \langle V,W,X\rangle =\arg\underset{V,W,X}{\text{min}}{\left\|\phi \left(A\right)-\phi \left(A\right)VX\right\|}_{F}^{2}+\alpha {\left\|H-WX\right\|}_{F}^{2}\\ \;+\;\beta {\left\|W\right\|}_{F}^{2}+\lambda_{1}{\left\|X\right\|}_{1}+\lambda_{2}\sum_{i=1}^{k}({\left\|X_{i}-M_{i}\right\|}_{F}^{2}\\ \;+\;{\left\|X_{i}\right\|}_{F}^{2}) \end{array}$$

The optimization process of Equation (8) has been discussed in our previous study (Wu et al., 2017). Then, the test sample *y* and dictionary *D* in Equation (4) can be replaced by $\phi \left(y\right)\in R^{d_{map}}$ and ϕ(*A*)*V* respectively as:

(9) $$\begin{array}{l} x=arg\;\underset{x}{\min}||\phi \left(y\right)-\phi \left(A\right)Vx|{|}_{2}^{2}+\lambda ||X|{|}_{1} \end{array}$$

where λ is a scalar constant as above.

Let $T\left(x\right)=\;\underset{x}{\text{min}}\;||\phi \left(y\right)-\phi \left(A\right)Vx|{|}_{2}^{2}$, then *T*(*x*) can be simplified as:

(10) $$\begin{array}{l} T\left(x\right)=\underset{x}{\text{min}}tr\left(x^{T}Px-2x^{T}Q+S\right) \end{array}$$

where *P* = *V*^*T*^*k*(*A, A*)*V*, *Q* = *V*^*T*^*k*(*y, A*), and *S* = *k*(*y, y*).

Using the conclusions in previous study (Harandi and Salzmann, 2015), Equation (10) is equivalent to:

(11) $$\begin{array}{l} T\left(x\right)=arg\;\underset{x}{\min}||ỹ-\tilde{D}x|{|}_{2}^{2} \end{array}$$

where $ỹ=\Sigma^{-\frac{1}{2}}U^{T}Q$, $\tilde{D}=\Sigma^{\frac{1}{2}}U^{T}$, and *U Σ U*^*T*^ is the SVD of *P* (Nguyen et al., 2012). Then Equation (9) can be denoted as:

(12) $$\begin{array}{l} \langle x\rangle =arg\;\underset{x}{\min}{||ỹ-\tilde{D}x|{|}_{2}^{2}+\lambda }_{1}||X|{|}_{1} \end{array}$$

The convex problems in Equation (12) can be efficiently solved by plenty of tools such as the *L*_1_-magic software package (Candes and Romberg, 2005), the GPSR package (Figueiredo et al., 2007) and the *L*_1_-homotopy package (Asif and Romberg, 2010).

Finally, the identification of the test sample *y* can be employed using Equation (5) as follows:

$$\begin{array}{l} label\left(y\right)=\arg\;\underset{l}{\text{max}}{\left\{W_{x}\right\}}_{l},l=1,2,\ldots ,k \end{array}$$

where the {·}_*l*_ represents the *l*-th element in the brace.

As it is shown in the MKL algorithm (Sonnenburg et al., 2006), suppose there are *J* features for each sample, the kernel can be combined by convex combinations of *J* kernels, i.e.,

(13) $$\begin{array}{l} k\left(x,y\right)=\overset{J}{\underset{j=1}{\sum }}w_{j}k_{j}\left(x,y\right)w_{j}\geq 0,\;\overset{J}{\underset{j=1}{\sum }}w_{j}=1 \end{array}$$

where each sub-kernel *k*_*j*_ corresponds to feature *j*.

So far, the kernels involved in the solution of Equation (12) can be replaced by Equation (13) for the multi-feature fusion of MKSCDDL. The combination coefficients can be simply set to be equal across all the features or optimized by cross-validation on the training samples. The sub-kernels can be selected from linear kernel, polynomial kernels, Gaussian kernels and sigmoid kernels etc. After the substitution of the kernels involved in the solution of Equation (12), MKSCDDL is realized (Wu et al., 2017).

### Experimental setting

In MKSCDDL model and the classification scheme, there are several parameters need to be set, including the parameter α for the classification error term, λ for the sparse coding term, λ_1_ for the sparsity term, and λ_2_ for the with-Class-similar term. Here, for simplify, α was set with α = 1 to make the contribution of the classification error equal (Xu et al., 2016). Furthermore, the parameter in the classification scheme λ made a little effect in the experimental results. So, λ was set with λ = 0.001 in the experiment. For the parameters in the optimization model λ_1_ and λ_2_, the optimal values were searched from a small set of {0.001, 0.005, 0.01, 0.05, 0.1} with a 5-fold cross-validation on the training set (Wu et al., 2017). For the AD and CU data set: λ_1_ = 0.001, λ_2_ = 0.1. For the MCI and CU data set: λ_1_ = 0.05, λ_2_ = 0.05. For the AD and MCI data set: λ_1_ = 0.05, λ_2_ = 0.005.

The dictionary size in MKSCDDL, mSCDDL, and SCDDL were set as 20 atoms (equivalent to 10 atoms for each class) for AD/CU, MCI/CU and AD/MCI classification; for MKL and JRC algorithms, all the training samples were trained for the model and classification; and for mSRC, all the training samples were used as a dictionary.

In this study, linear kernel was employed for MKSCDDL in the experiment. The combining weight parameters of three modalities for MKSCDDL was derived based on grid search approach with the range of [0,1] at a step size of 0.1 with a 5-fold cross-validation on training set (Zhang et al., 2011; Xu et al., 2015, 2016). Particularly, the combing weight parameters optimized corresponding to sMRI, FDG-PET and florbetapir-PET for classifying AD from CU are 0.5, 0.3, and 0.2; for discriminating MCI from CU are 0.2, 0.7, and 0.1; for detecting MCI from AD are 0.3, 0.6, and 0.1.

To evaluate the performance of all competing methods, their accuracy (the ratio of samples correctly classified among the test samples), sensitivity (the ratio of positive classes that were correctly identified), specificity (the ratio of negative classes that were accurately classified), and the areas under the Receiver Operating Characteristic (ROC) curves (AUC) were employed and compared in classification. For each group (AD, MCI, and CU), samples (subjects) were divided randomly into training and test sets. Sixty samples were selected randomly as the training set, and the rest comprised the test set. The division process was then repeated five times for the results of means and standard deviations, which were reported in this paper. Then, a two-sample t-test was carried out for each comparison pair to obtain the *p*-value.

In order to find the biomarkers for AD, MCI and CU classification, the 90 features were ranked according to the significance of the two-sample *t*-test. Then, the classification accuracy with different number (from 1 to 90) of the ranked 90 features has been calculated based on MKSCDDL (Zhang et al., 2011; Xu et al., 2016).

## Results and discussions

### Comparison with single-modality SCDDL

**Figure 1:**
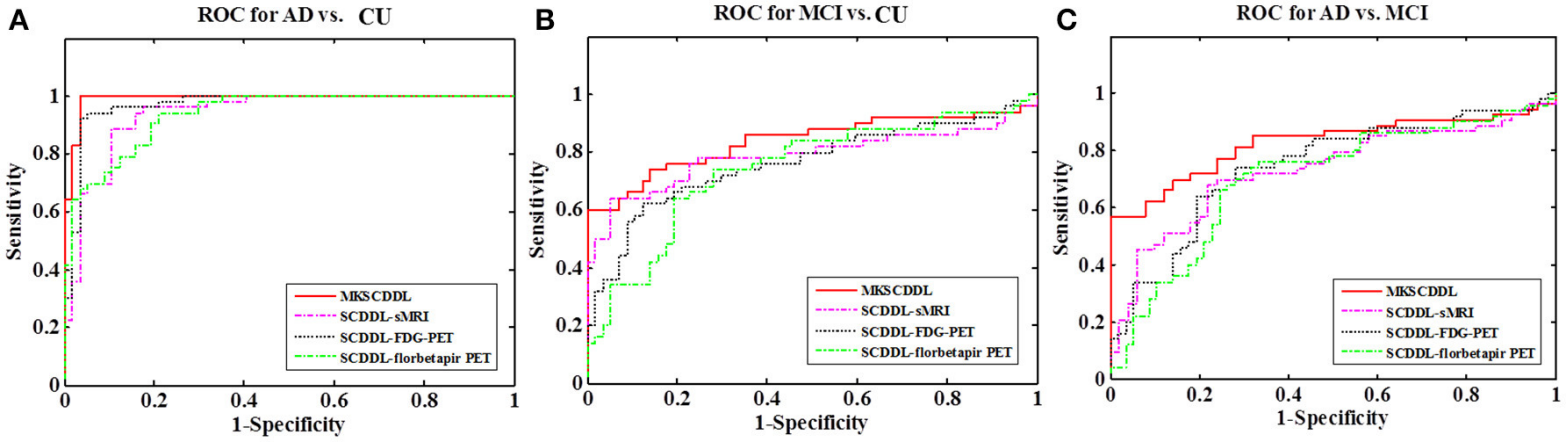
Comparison of the ROC curves based on SCDDL-sMRI, SCDDL-FDG-PET, SCDDL-florbetapir-PET, and MKSCDDL **(A)** for classification AD and CU; **(B)** for classification MCI and CU; and **(C)** for classification AD and MCI.

**Figure 2:**
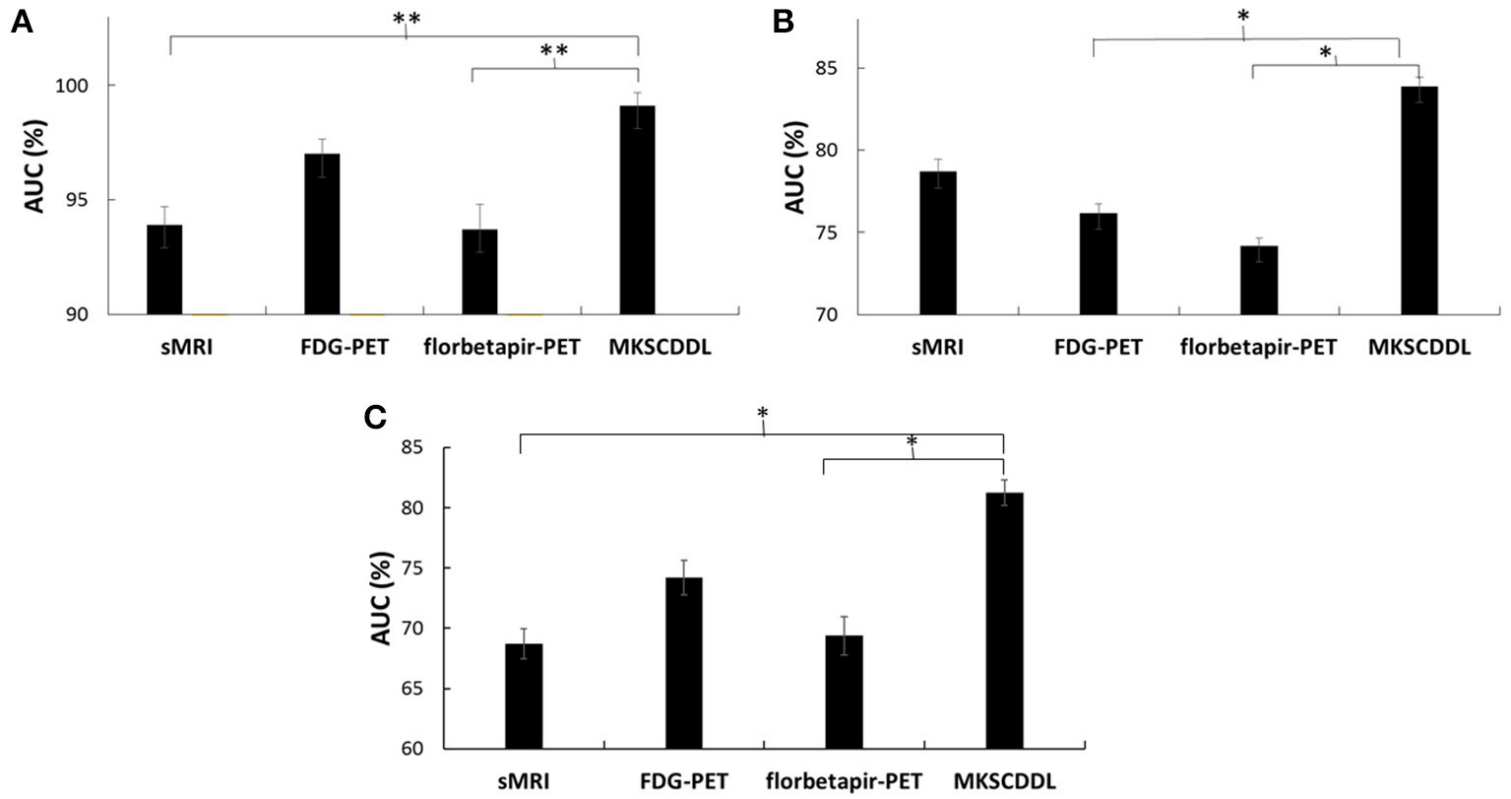
Comparison of the areas under the ROC curves based on SCDDL-sMRI, SCDDL-FDG-PET, SCDDL-florbetapir-PET, and MKSCDDL **(A)** for classification AD and CU; **(B)** for classification MCI and CU; and **(C)** for classification AD and MCI (^**^indicates 0.01 ≤ *p* < 0.05; ^*^indicates 0.05 ≤ *p* < 0.10).

The performance of using single-modality SCDDL (SCDDL-sMRI, SCDDL-FDG-PET, and SCDDL-florbetapir-PET) and MKSCDDL (sMRI + FDG-PET + florbetapir-PET) were evaluated, as shown in Figures 1, 2 and Table 2, the MKSCDDL achieved higher accuracy in classifying AD, MCI, and CU than single-modality SCDDL methods.

**Table 2:** Comparison of the performance of single-modality (SCDDL-sMRI, SCDDL-FDG-PET, and SCDDL-florbetapir-PET) and multi-modality methods based on MKSCDDL in classification AD, CU; MCI, CU; and AD, MCI.

**Algorithm**	**AD vs. CU (%)**				**MCI vs. CU (%)**				**AD vs. MCI (%)**			
	**ACC**	**SE**	**SP**	**AUC**	**ACC**	**SE**	**SP**	**AUC**	**ACC**	**SE**	**SP**	**AUC**
SCDDL-sMRI	88.27 ± 1.51	94.53 ± 1.88	82.46 ± 1.85	93.90 ± 0.80	71.96 ± 1.08	69.80 ± 1.63	72.11 ± 1.54	78.70 ± 0.76	63.69 ± 3.45	60.85 ± 2.85	65.21 ± 2.58	68.70 ± 1.26
SCDDL-FDG-PET	91.18 ± 1.72	86.42 ± 1.64	95.61 ± 1.24	97.00 ± 0.65	72.50 ± 1.24	62.20 ± 1.35	**81.23 ± 1.81**	76.20 ± 0.53	72.23 ± 2.85	65.20 ± 2.95	75.60 ± 3.23	74.20 ± 1.44
SCDDL-florbetapir-PET	85.64 ± 1.87	85.51 ± 1.95	85.61 ± 1.29	93.70 ± 1.09	70.09 ± 0.89	66.00 ± 0.97	73.68 ± 1.02	74.20 ± 0.45	63.50 ± 3.71	64.00 ± 2.96	71.64 ± 3.02	69.40 ± 1.58
MKSCDDL	**98.18 ± 0.29**	**99.81 ± 0.35**	**96.49 ± 0.41**	**99.10 ± 0.60**	**78.50 ± 0.39**	**76.00 ± 0.42**	81.06 ± 0.48	**83.90 ± 0.55**	**74.47 ± 1.02**	**72.44 ± 1.53**	**78.99 ± 1.52**	**79.10 ± 1.70**

*AD, Alzheimer's disease; CU, cognitively unimpaired; MCI, Mild Cognitive Impairment; ACC, classification accuracy; SE, classification sensitivity; SP, classification specificity; AUC, the area under the ROC curve. All the results had 5 folder cross-validations. The bold values mean the best performance in the corresponding column*.

For discriminating AD from CU, MKSCDDL achieved an accuracy of 98.18% (with 99.81% sensitivity and 96.49% specificity) that was much better than the best accuracy of 91.18% with single-modality method (using SCDDL-FDG-PET). Further, the comparison of the ROC curves for classification of AD and CU is shown in Figure 1A, and the comparison of AUCs is shown in Table 2. The ROC curve of MKSCDDL was closer to the top-left corner than that of SCDDL-FDG-PET, SCDDL-florbetapir-PET, and SCDDL-sMRI. The AUC of MKSCDDL was 0.991, which was better than the single-modality methods (AUC = 0.939, *p* = 0.046 for SCDDL-sMRI; AUC = 0.937, *p* = 0.028 for SCDDL-florbetapir-PET; and AUC = 0.970, *p* = 0.151 for SCDDL-FDG-PET, which was not significant in validation, but was numerically greater) as shown in Figure 2A.

For classifying MCI from CU, MKSCDDL achieved an accuracy of 78.50% (with sensitivity of 76.00% and specificity of 81.06%), which was greater than all three single-modality methods (the best classification accuracy was 72.50% when using SCDDL-FDG-PET). The comparison of the ROC curves for classification of MCI and CU are shown in Figure 1B and the comparison of AUCs is shown in Table 2. The ROC curve of MKSCDDL was closer to the top-left corner than that of SCDDL-sMRI, SCDDL-florbetapir-PET, and SCDDL-FDG-PET. Further, based on the significance validation, MKSCDDL was significantly much better than the single-modality methods with AUC, which was 0.839 for the multi-modality method compared with that of the single-modality methods (AUC = 0.762, *p* = 0.094 for SCDDL-FDG-PET; AUC = 0.742, *p* = 0.076 for SCDDL-florbetapir-PET; AUC = 0.787, *p* = 0.315 for SCDDL-sMRI, which were numerically better, though were not significant in validation) as shown in Figure 2B.

For classifying AD from MCI, MKSCDDL achieved an accuracy of 74.47% (with sensitivity of 72.44% and specificity of 78.99%), which was greater than all three single-modality methods (the best classification accuracy was 72.23% when using SCDDL-FDG-PET). The comparison of the ROC curves for classification of AD and MCI are shown in Figure 1C and the comparison of AUCs is shown in Table 2. The ROC curve of MKSCDDL was closer to the top-left corner than that of SCDDL-sMRI, SCDDL-florbetapir-PET, and SCDDL-FDG-PET. Further, based on significant validation, MKSCDDL was significantly much better than the single-modality methods with AUC, which was 0.791 for the multi-modality method compared with that of the single-modality methods (AUC = 0.687, *p* = 0.091 for SCDDL-sMRI; AUC = 0.694, *p* = 0.107 for SCDDL-florbetapir-PET; and AUC = 0.742, *p* = 0.198 for SCDDL-FDG-PET, which was numerically better, though were not significant in validation) as shown in Figure 2C.

The MKSCDDL achieved better classification accuracy and AUC for AD, MCI, and CU classification than the methods based on single-modality SCDDL (SCDDL-sMRI, SCDDL-FDG-PET, and SCDDL-florbetapir-PET), as seen in the results above, either statistically or numerically. The results we derived here were also consistent with those of other studies that have reported fusing multiple modalities could obtain better classification accuracy (Zhang et al., 2011; Westman et al., 2012; Xu et al., 2016).

Notably, on differentiating between MCI and CU, the classification specificity based on SCDDL-FDG-PET was 81.23%, which was slightly higher than that based on MKSCDDL (81.06%), whereas the classification sensitivity based on SCDDL-FDG-PET (62.20%) was much lower than that of MKSCDDL (76.00%). Lower sensitivity with only marginally higher specificity (which could be due to random noise) would result in underdiagnosis. The MKSCDDL method had higher sensitivity and outstanding specificity that was comparable with that of SCDDL-FDG-PET, and much higher than that of the other methods. Therefore, the results suggest the feasibility of using MKSCDDL for neuroimaging classification tasks. These meant that the MKSCDDL method was much or slightly better than SCDDL-florbetapir-PET, SCDDL-sMRI and SCDDL-FDG-PET in differentiating AD or MCI from CU.

### Comparison with several other multi-modality methods

**Figure 3:**
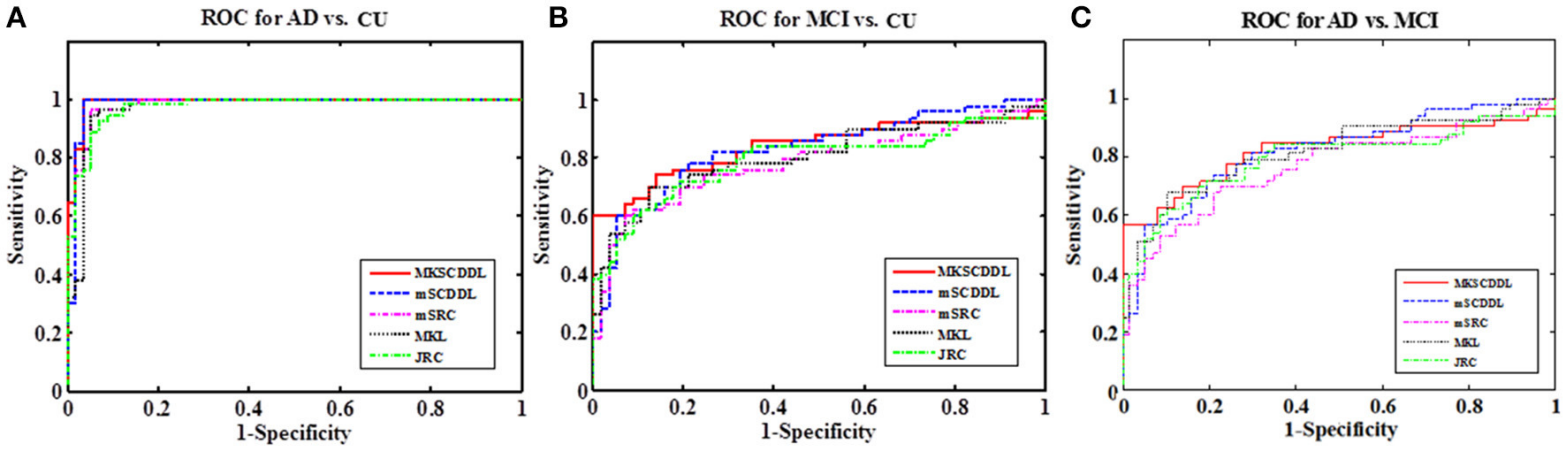
Comparison of the ROC curves based on JRC, MKL, mSRC, mSCDDL, and MKSCDDL **(A)** for classification AD and CU; **(B)** for classification MCI and CU; and **(C)** for classification AD and MCI.

**Figure 4:**
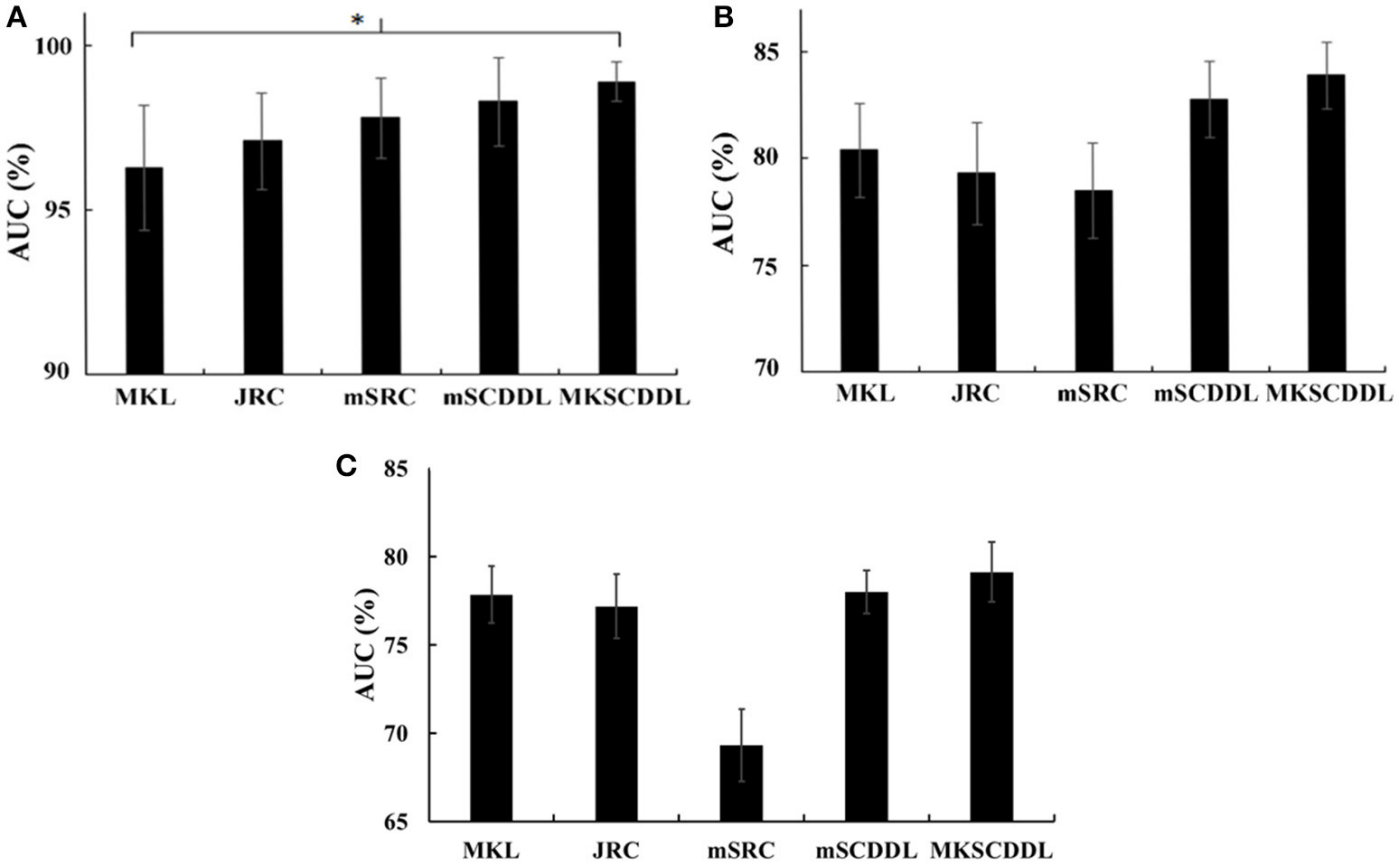
Comparison of the areas under the ROC curves based on MKL, JRC, mSRC, mSCDDL, and MKSCDDL **(A)** for classification AD and CU; **(B)** for classification MCI and CU; and **(C)** for classification AD and MCI (^*^indicates 0.05 ≤ *p* < 0.10).

**Figure 5:**
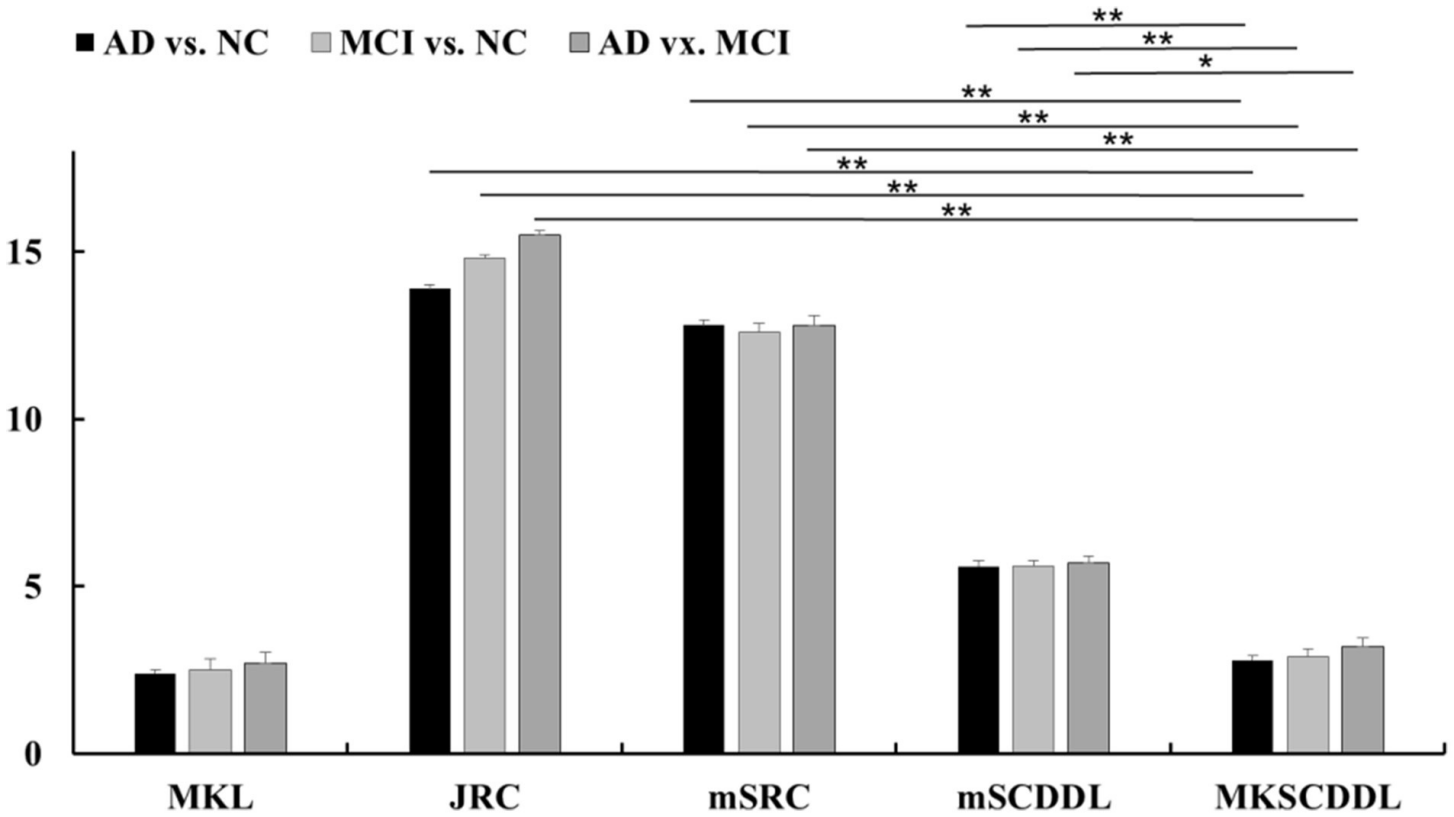
Comparison of testing time of different multi-modality methods for classification AD, MCI, and CU based on MKL, JRC, mSRC, mSCDDL, and MKSCDDL (^**^indicates 0.01≤ *p* < 0.05; ^*^indicates 0.05 ≤ *p* < 0.10).

The performance of using MKL, JRC, mSRC, mSCDDL, and MKSCDDL were evaluated and compared, including recognition rate, ROC curve and testing time. As shown in Figures 3–**5** and Table 3, the MKSCDDL achieved higher accuracy in classifying AD or MCI from CU than other multimodal methods, and outperforms in testing time.

**Table 3:** Comparison of the performance of MKL, JRC, mSRC, mSCDDL, and MKSCDDL in classification AD, CU; MCI, CU; and AD, MCI.

**Algorithm**	**AD vs. CU (%)**				**MCI vs. CU (%)**				**AD vs. MCI (%)**			
	**ACC**	**SE**	**SP**	**AUC**	**ACC**	**SE**	**SP**	**AUC**	**ACC**	**SE**	**SP**	**AUC**
MKL	93.64 ± 0.87	96.23 ± 0.69	91.23 ± 1.07	96.30 ± 1.90	74.77 ± 0.74	74.00 ± 1.01	75.44 ± 1.14	80.40 ± 1.03	72.94 ± 1.87	72.04 ± 1.56	74.10 ± 1.95	77.90 ± 1.60
JRC	94.55 ± 1.18	98.11 ± 1.85	91.23 ± 1.42	97.10 ± 1.45	73.83 ± 1.02	72.00 ± 1.40	75.44 ± 1.25	79.30 ± 1.77	72.05 ± 1.98	70.68 ± 2.03	73.23 ± 2.23	77.20 ± 1.83
mSRC	94.55 ± 1.35	96.23 ± 1.64	92.98 ± 1.57	97.80 ± 1.92	75.70 ± 1.44	66.00 ± 1.67	**84.21 ± 2.12**	78.50 ± 2.04	68.55 ± 2.01	64.26 ± 2.44	74.66 ± 2.54	69.30 ± 2.03
mSCDDL	97.36 ± 1.00	99.25 ± 1.32	95.61 ± 1.49	98.50 ± 1.33	77.66 ± 1.12	75.00 ± 1.46	80.70 ± 1.29	82.80 ± 1.31	73.20 ± 1.00	69.31 ± 1.85	75.62 ± 1.61	78.00 ± 1.22
MKSCDDL	**98.18 ± 0.29**	**99.81 ± 0.35**	**96.49 ± 0.41**	**99.10 ± 0.60**	**78.50 ± 0.39**	**76.00 ± 0.42**	81.06 ± 0.48	**83.90 ± 0.55**	**74.47 ± 1.02**	**72.44 ± 1.53**	**78.99 ± 1.52**	**79.10 ± 1.70**

*The bold values mean the best performance in the corresponding column*.

For differentiating AD from CU, MKSCDDL achieved an accuracy of 98.18% accuracy CU that was higher than MKL (93.64%), JRC (94.55%), mSRC (94.55%), and mSCDDL (97.36%). The comparison of the ROC curves for classification of AD and CU is shown in Figure 3A and the comparison of AUCs is shown in Table 3. The ROC curve of MKSCDDL was closer to the top-left corner than that of MKL, JRC, mSRC, and mSCDDL. The areas under the ROC curves for differentiation of AD and CU based on the five different methods are displayed in Figure 4A, in which the MKSCDDL method (AUC = 0.991) performed equally well statistically or numerically better than the other three multi-modality methods (AUC = 0.963, *p* = 0.095 for MKL; AUC = 0.971, *p* = 0.291 for JRC; AUC = 0.978, *p* = 0.429 for mSRC; and AUC = 0.985, *p* = 0.603 for mSCDDL). Figure 5 has shown the computational time for classification of per test sample with the corresponding methods. As shown, MKSCDDL consumed much less testing time than JRC (*p* = 0.007), mSRC (*p* = 0.010), and mSCDDL (*p* = 0.036), and was comparable with the MKL (*p* = 0.208) method.

For classifying MCI from CU, MKSCDDL achieved an accuracy of 78.50% (with sensitivity of 76.00% and specificity of 81.06%), which was greater than MKL (74.77%), JRC (73.83%), mSRC (75.70%), and mSCDDL (77.66%). The comparison of the ROC curves for classification of MCI and CU are shown in Figure 3B and the comparison of AUCs is shown in Table 3. The ROC curve of MKSCDDL was closer to the top-left corner than that of MKL, JRC, mSRC, and mSCDDL. Further, based on significant validation, MKSCDDL was numerically better than the corresponding methods with AUC, which was 0.839 for the MKSCDDL method compared with that of the corresponding methods (AUC = 0.804, *p* = 0.534 for MKL; AUC = 0.793, *p* = 0.331 for JRC; AUC = 0.785, *p* = 0.223 for mSRC; and AUC = 0.828, *p* = 0.843 for mSCDDL), as shown in Figure 4B. As shown in Figure 5, MKSCDDL consumed much less testing time than JRC (*p* = 0.009), mSRC (*p* = 0.015) and mSCDDL (*p* = 0.047), and was comparable with the MKL (*p* = 0.389) method.

For classifying AD from MCI, MKSCDDL achieved an accuracy of 74.47% (with sensitivity of 72.44% and specificity of 78.99%), which was greater than MKL (72.94%), JRC (72.05%), mSRC (68.55%), and mSCDDL (73.20%). The comparison of the ROC curves for classification of AD and MCI are shown in Figure 3C and the comparison of AUCs is shown in Table 3. The ROC curve of MKSCDDL was closer to the top-left corner than that of MKL, JRC, mSRC, and mSCDDL. Further, based on significant validation, MKSCDDL was numerically better than the corresponding methods with AUC, which was 0.791 for the MKSCDDL method compared with that of the corresponding methods (AUC = 0.779, *p* = 0.600 for MKL; AUC = 0.772, *p* = 0.477 for JRC; AUC = 0.693, *p* = 0.120 for mSRC; and AUC = 0.780, *p* = 0.593 for mSCDDL), which shown in Figure 4C. As shown in Figure 5, MKSCDDL consumed much less testing time than JRC (*p* = 0.011), mSRC (*p* = 0.019) and mSCDDL (*p* = 0.059), and was comparable with the MKL (*p* = 0.352) method.

### Biomarkers for AD, MCI, and CU classification

**Figure 6:**
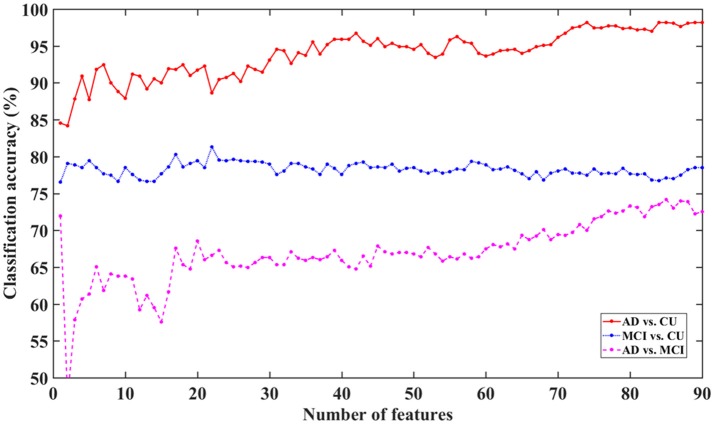
Classification accuracy for AD, MCI, and CU with different feature dimensions based on MKSCDDL.

To characterize the classification performance for AD, MCI, and CU with all 90 features (without feature selection), the classification accuracy has been investigated under feature selection with 1, 2, 3, …, or 90 features for each of the ranked 90 features. The results of classification performance for different numbers of ranked features are shown in Figure 6.

The figure shows that the MKSCDDL method could reach strong classification accuracy even with fewer than 5 features (the top 5% ranked features on sMRI, FDG-PET, and florbetapir-PET) for AD/MCI/CU classification. In particular, there was higher than 90% accuracy for classifying AD from CU, higher than 78% accuracy for distinguishing MCI from CU, and higher than 61% accuracy for discriminating AD and MCI. The MKSCDDL method was stable (with less ups and downs) for the classification of AD/MCI from CU, which indicated that redundant features likely introduced little interference of classification. For classification of AD and MCI, though the accuracy was also acceptable, it was not as stable as the classification accuracy for AD/MCI with CU, which may be due to the biomarkers for AD and MCI having very high similarity. When the top 10% features were used, the accuracy for classification of AD and MCI was higher than 64%.

As shown in Figure 6, the MKSCDDL could achieve a promising or acceptable accuracy even with less than 5 features (the top 5% ranked features). Thus, for convenience, one could apply a small set of features to effectively discriminate AD, MCI, and CU. Here, the top 5–10% ranked features (4–9 features) consisted of sMRI, FDG-PET, and florbetapir-PET data and could be chosen as biomarkers for further classification (Xu et al., 2016).

**Figure 7:**
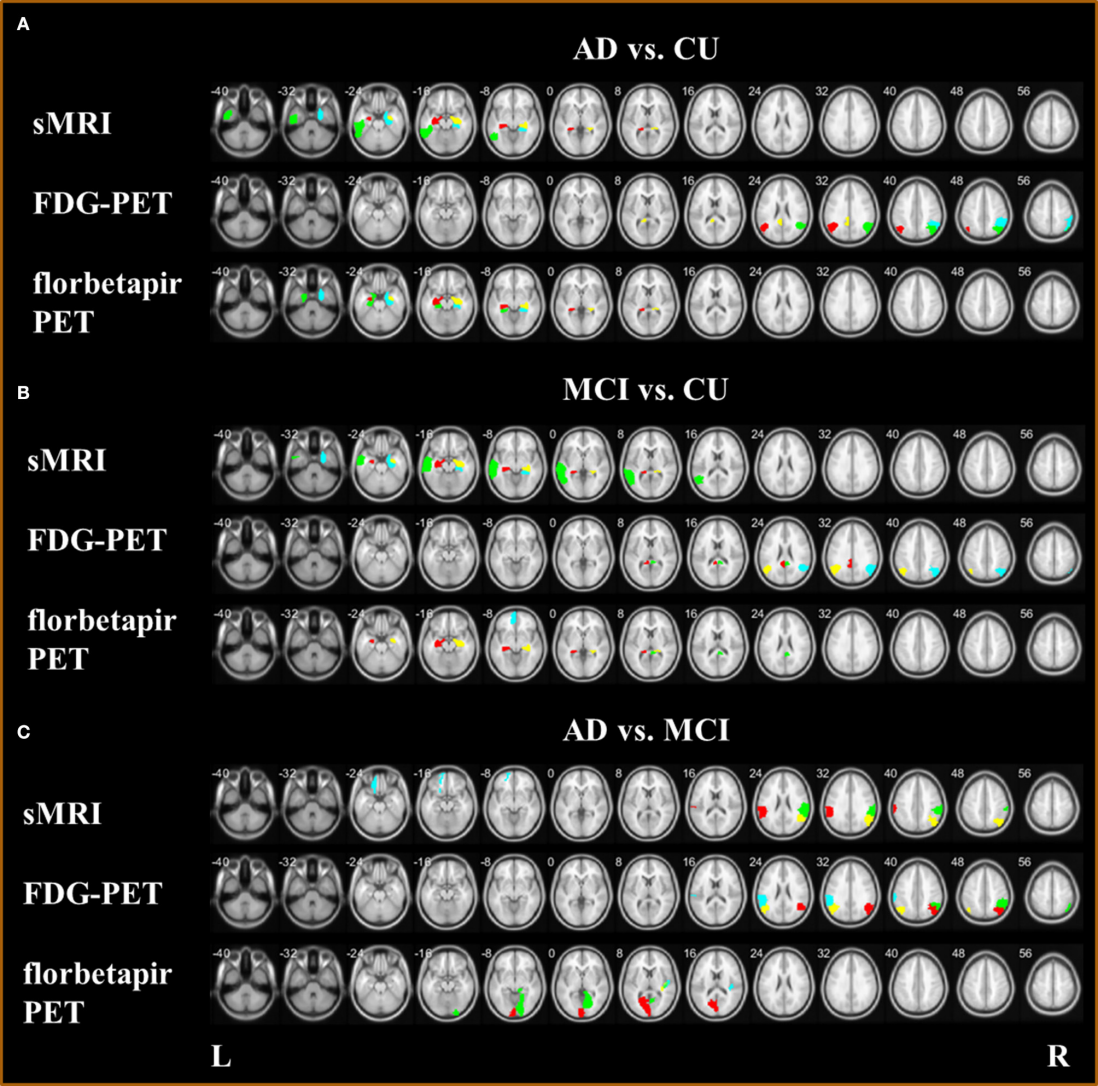
Biomarkers with sMRI, FDG-PET, and florbetapir-PET **(A)** for classification AD and CU; **(B)** for classification MCI and CU; and **(C)** for classification AD and MCI.

The biomarkers of different modalities for classification of the AD, MCI, and CU groups are displayed in Table 4 and Figure 7. For classification of AD and CU, the Hippocampus, Inferior Temporal, and ParaHippocampal may be the discriminating biomarkers on sMRI; the Angular, Posterior Cingulum, and Inferior Parietal may be the important regions on FDG-PET; and the Hippocampus and ParaHippocampal may be the key regions on florbetapir-PET. For discriminating MCI from CU, the Hippocampus, Middle Temporal, and ParaHippocampal may be the discriminating biomarkers on sMRI; the Angular and Posterior Cingulum may be the important regions on FDG-PET; and the Hippocampus, Posterior Cingulum, and Middle Frontal (Orbital part) may be the key regions on florbetapir-PET. For differentiating AD and MCI, the SupraMarginal, Angular, and left Superior Frontal (Orbital part) were the discriminating biomarkers on sMRI; the Angular, Inferior Parietal, and SupraMarginal may be the important regions on FDG-PET; and the Calcarine, Heschl, and Lingual may be the key regions on florbetapir-PET.

**Table 4:** The most discriminating regions for classification AD, MCI, and CU based on sMRI, FDG-PET, and florbetapir-PET.

**sMRI**	**FDG-PET**	**Florbetapir PET**
**AD vs. CU**		
Left hippocampus	Left angular	Left hippocampus
Right hippocampus	Left posterior cingulum	Right hippocampus
Left inferior temporal	Right angular	Left parahippocampal
Right parahippocampal	Right inferior parietal	Right parahippocampal
**MCI vs. CU**		
Left hippocampus	Left posterior cingulum	Left hippocampus
Right hippocampus	Left angular	Right hippocampus
Left middle temporal	Right posterior cingulum	Right posterior cingulum
Right parahippocampal	Right angular	Left middle frontal (orbital part)
**AD vs. MCI**		
Left supramarginal	Right angular	Left calcarine
Right angular	Left angular	Left heschl
Right supramarginal	Right inferior parietal	Right lingual
Left superior frontal (orbital part)	Left supramarginal	Right heschl

For AD and CU classification, the Hippocampus (Wisse et al., 2014; de Flores et al., 2015; Voineskos et al., 2015), Inferior Temporal (Seo et al., 2017), ParaHippocampal (Guo et al., 2014; Peng et al., 2016), Angular (Sanabria-Diaz et al., 2013), Posterior Cingulum (Nakata et al., 2009; Demirhan et al., 2015), and Inferior Parietal (Murray et al., 2015; Zhang et al., 2015) have been proposed in several studies to be effective biomarkers. The Hippocampus (Wee et al., 2011; Zhou et al., 2011; Liu et al., 2014a), Middle Temporal (Lenzi et al., 2011; Jiang et al., 2014), ParaHippocampal (Cerami et al., 2015; Kato et al., 2016), Angular (Nobili et al., 2010; Martlno et al., 2013; Zu et al., 2015), Posterior Cingulum (Choo et al., 2010; Yu et al., 2017), and Middle Frontal (Orbital part) (Xiang et al., 2013) have been reported as the important regions for discriminating MCI and CU. For differentiating AD and MCI, the SupraMarginal (Esposito et al., 2013; Moretti, 2015), Angular (Hirao et al., 2005; Griffith et al., 2010; Li et al., 2016), Superior Frontal (Orbital part) (Liu et al., 2012), Inferior Parietal (Desikan et al., 2009; Triplett et al., 2016), Calcarine (Liu et al., 2012), Heschl (Hanggi et al., 2011), and Lingual (Li et al., 2016) may be the key biomarkers for diagnosis.

Therefore, MKSCDDL was proved as a very efficient method for classifying AD or MCI from CU, and had potential to discriminate AD from MCI, as compared to the single-modality method and several state-of-art multi-modality methods. The MKSCDDL method performed better than MKL, JRC, mSRC, and mSCDDL in terms of accuracy rate and AUC, often significantly on validation but at least numerically for AD, MCI, and CU classification. In addition, the MKSCDDL method took less computation time than did JRC, mSRC, and mSCDDL, and was comparable to MKL in terms of computation time. Together, this indicates that the MKSCDDL method could potentially play an important role in AD and MCI diagnosis.

## Conclusions

In this study, a novel DL method, named as MKSCDDL with previous successful application to face recognition, was introduced combining sMRI, FDG-PET, and florbetapir-PET for differentiating AD, MCI, and CU. The results suggested that the MKSCDDL is promising for classification and diagnose diseases with neuroimaging data.

## Ethics statement

All procedures performed in studies involving human participants were in accordance with the ethical standards of the institutional and/or national research committee and with the 1964 Helsinki declaration and its later amendments or comparable ethical standards.

### Informed consent

Informed consent was obtained from all individual participants included in the study.

## Author contributions

XW, LY: designed the study. LX, KC: collected the original imaging data. QL, XW, KC: managed and analyzed the imaging data. QL and XW: wrote the manuscript. All authors contributed to and have approved the final manuscript.

### Conflict of interest statement

The authors declare that the research was conducted in the absence of any commercial or financial relationships that could be construed as a potential conflict of interest.
