# Supplementary material for: Classification of Alzheimer's Disease, Mild Cognitive Impairment, and Cognitively Unimpaired Individuals Using Multi-feature Kernel Discriminant Dictionary Learning
Source: Front Comput Neurosci. 2018 Jan 9;11:117. doi: 10.3389/fncom.2017.00117 (PMC5767247; doi:10.3389/fncom.2017.00117)
Supplement: Supplementary file 1 [file Table1.docx]

**Table S1.** Regions of Interest in the AAL-atlas.

| **Regions** | **Regions** |
| --- | --- |
| left Amygdala | right Inferior Occipital |
| right Amydala | left Middle Occipital |
| left Angular | right Middle Occipital |
| right Angular | left Superior Occipital |
| left Calcarine | right Superior Occipital |
| right Calcarine | left Olfactory |
| left Caudate | right Olfactory |
| right Caudate | left Pallidum |
| left Anterior Cingulum | right Pallidum |
| right Anterior Cingulum | left Paracentral Lobule |
| left Middle Cingulum | right Paracentral Lobule |
| right Middle Cingulum | left ParaHippocampal |
| left Posterior Cingulum | right ParaHippocampal |
| right Posterior Cingulum | left Inferior Parietal |
| left Cuneus | right Inferior Parietal |
| right Cuneus | left Superior Parietal |
| left Inferior Frontal (Opercular part) | right Superior Parietal |
| right Inferior Frontal (Opercular part) | left Postcentral |
| left Inferior Frontal (Orbital part) | right Postcentral |
| right Inferior Frontal (Orbital part) | left Precentral |
| left Inferior Frontal (Triangular part) | right Precentral |
| right Inferior Frontal (Triangular part) | left Precuneus |
| left Medial Frontal (Orbital part) | right Precuneus |
| right Medial Frontal (Orbital part) | left Putamen |
| left Middle Frontal | right Putamen |
| right Middle Frontal | left Rectus |
| left Middle Frontal (Orbital part) | right Rectus |
| right Middle Frontal (Orbital part) | left Rolandic (Opercular part) |
| left Superior Frontal | right Rolandic (Opercular part) |
| right Superior Frontal | left Supplementary Motor Area |
| left Superior Frontal (Medial part) | right Supplementary Motor Area |
| right Superior Frontal (Medial part) | left SupraMarginal |
| left Superior Frontal (Orbital part) | right SupraMarginal |
| right Superior Frontal (Orbital part) | left Inferior Temporal |
| left Fusiform | right Inferior Temporal |
| right Fusiform | left Middle Temporal |
| left Heschl | right Middle Temporal |
| right Heschl | left Middle Temporal (Polo part) |
| left Hippocampus | right Middle Temporal (Polo part) |
| right Hippocampus | left Superior Temporal (Polo part) |
| left Insula | right Superior Temporal (Polo part) |
| right Insula | left Superior Temporal |
| left Lingual | right Superior Temporal |
| right Lingual | left Thalamus |
| left Inferior Occipital | right Thalamus |
